# Potential loss of revenue due to errors in clinical coding during the implementation of the Malaysia diagnosis related group (MY-DRG^®^) Casemix system in a teaching hospital in Malaysia

**DOI:** 10.1186/s12913-018-2843-1

**Published:** 2018-01-25

**Authors:** S. A. Zafirah, Amrizal Muhammad Nur, Sharifa Ezat Wan Puteh, Syed Mohamed Aljunid

**Affiliations:** 1 Faculty of Medicine, National University of Malaysia, International Centre for Casemix and Clinical Coding, UKM Medical Centre, Bandar Tun Razak, 56000 Kuala Lumpur, Cheras Malaysia; 2 United Nations University – International Institute for Global Health, UKM Medical Centre, Bandar Tun Razak, 56000 Kuala Lumpur, Cheras Malaysia; 3 Department of Health Policy and Management, Faculty of Public Health, Kuwait University, P.O Box 24923, 13110 Kuwait, Safat Kuwait

**Keywords:** Diagnosis coding, Procedure coding, Clinical coding, Coding error, Casemix system, DRG, My-DRG®

## Abstract

**Background:**

The accuracy of clinical coding is crucial in the assignment of Diagnosis Related Groups (DRGs) codes, especially if the hospital is using Casemix System as a tool for resource allocations and efficiency monitoring. The aim of this study was to estimate the potential loss of income due to an error in clinical coding during the implementation of the Malaysia Diagnosis Related Group (MY-DRG^®^) Casemix System in a teaching hospital in Malaysia.

**Methods:**

Four hundred and sixty-four (464) coded medical records were selected, re-examined and re-coded by an independent senior coder (ISC). This ISC re-examined and re-coded the error code that was originally entered by the hospital coders. The pre- and post-coding results were compared, and if there was any disagreement, the codes by the ISC were considered the accurate codes. The cases were then re-grouped using a MY-DRG^®^ grouper to assess and compare the changes in the DRG assignment and the hospital tariff assignment. The outcomes were then verified by a casemix expert.

**Results:**

Coding errors were found in 89.4% (415/424) of the selected patient medical records. Coding errors in secondary diagnoses were the highest, at 81.3% (377/464), followed by secondary procedures at 58.2% (270/464), principal procedures of 50.9% (236/464) and primary diagnoses at 49.8% (231/464), respectively. The coding errors resulted in the assignment of different MY-DRG^®^ codes in 74.0% (307/415) of the cases. From this result, 52.1% (160/307) of the cases had a lower assigned hospital tariff. In total, the potential loss of income due to changes in the assignment of the MY-DRG^®^ code was RM654,303.91.

**Conclusions:**

The quality of coding is a crucial aspect in implementing casemix systems. Intensive re-training and the close monitoring of coder performance in the hospital should be performed to prevent the potential loss of hospital income.

## Background

Demographic shifts such as extended longevity and increases in lifestyle diseases specifically non-communicable diseases have caused Malaysia’s healthcare costs to escalate dramatically. In 2013, the government spending in the health care sector was only 4.5% (RM 44,748 million) of the nation’s gross domestic product (GDP). The Malaysia Health System Review reported that Malaysia’s health care expenditures were in the middle range for high and middle-income countries in the Asian region [1]. Thus the management of these resources must be performed wisely to ensure that their distribution of the resources is sufficient and fairly distributed to the entire population. Republic of Indonesia, for example, the nation implemented Social Health Insurance in 2004 to meet their goal of universal health coverage and to ensure fairness in health care financing [2]. Malaysia may also need to follow in their neighboring country’s footsteps for better management of their health care resources. This health reform is achievable by using the Casemix System.

The Casemix System is a patient classification system that classifies patients with similar clinical characteristics into one single homogeneous costing group. This system was developed by Bob Fetter and John Thompson from Yale University in 1976. The Diagnosis Related Groups (DRGs) was the first grouping system developed under this system, and it was used as a mean of relating the type of patients that a hospital treats to the cost incurred by the hospital [3, 4]. In health system’s management, DRGs is one of the powerful tools that can be used by hospital managers. DRG is not only usable for measuring hospital costs but also for evaluating hospital performance [5]. This system has been adopted by various countries, and each one has its own version of DRG, such as Germany (G-DRG), the United Kingdom (Healthcare Resources Groups; HRG), Australia (Australia Refined Diagnostic Group; AR-DRG), and Malaysia (Malaysia Diagnostic Related Group; MY-DRG ®) [6–8]. DRG codes are derived from a coding process, which is one of the essential elements besides costing that is used in the implementation of the casemix system as the provider payment tools.

The coding process is an assignment of numeric and alphanumeric digits and characters to specific diagnostic and procedural phrases. The coding process involved two essential components: diagnosis coding and procedure coding [9]. In Malaysia’s system, codes for diagnostic phrases are assigned on the basis of the International Classification of Disease 10th Revision (ICD 10), and the codes for the procedural phrases are assigned on the basis of the International Classification of Disease, Ninth Clinical Modification (ICD-9-CM). ICD 10 comprises guidelines for recording and coding, and it classifies disease into 24 chapters. The ICD 10 includes three volumes: Volume 1 (primary classification), Volume 2 (ICD user guidance) and Volume 3 (alphabetical index to the classification). On the other hand, ICD-9-CM also comprises three volumes, which are Volume 1 (a tabular listing of diseases), Volume 2 (an alphabetical listing of diseases) and Volume 3 (a numerical and alphabetical listing of surgical or non-surgical procedures that may be performed by physicians).

**Fig. 1:**
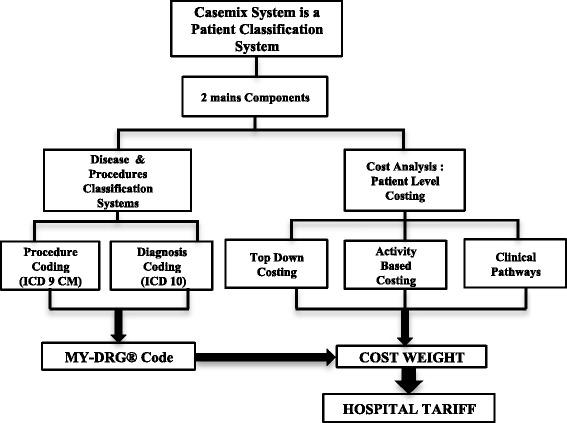
Flow of Casemix System. Casemix is a patient classification system where there were two main components namely Clinical Coding and Cost Analysis. The coding process is conducted according to the International Classification Disease 10th Revision (ICD 10) for diagnosis coding and International Classification of Disease Ninth Clinical Modification (ICD-9 CM) for procedure coding. On the other hand, costing methods that are usually employed in Casemix System are Top Down Costing and Activity Based Costing. In Casemix system, the information from the clinical coding process is used to generate the MY-DRG® Code, and these codes will be assigned to a hospital tariff according to the cost weight calculated at the cost analysis part

Coding information provides a rich database that can be utilized for epidemiological studies, clinical research, hospital audits, health care funding distribution and developing healthcare policies. In the Casemix System, DRGs codes are made up from a combination of diagnosis and procedure codes. Together with patients’ demographics information, both the diagnosis and procedures codes will be consolidated in a software called grouper. The grouper will then extract the DRGs codes based on the combination of these information. These codes will then be used during the assignment of the hospital tariffs, which are calculated using the cost weight developed in the cost analysis part (Fig. 1).

**Fig. 2:**
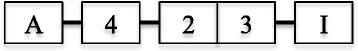
Structure of MY-DRG^®^ Code. “A” indicates the CMG Group. “4” indicates the discipline. “23” indicates the DRG Code. “I” indicates the severity level of the patient

MY-DRG® grouper is one of the grouper that are being used in Malaysia. In this system, each DRGs code was made up from 5 alphanumeric codes (one letter and four numbers). The first digit refers to the Casemix Main Group (CMG) which refers to the body systems (labeled in Alphabet (A-Z), for example, A = Infectious and parasitic disease group), the second digit refers to the discipline to which the patients are assigned to (1 = surgical, 4 = medical, 6 = Obstetric & Gynecology, 8 = Paediatric), the third and fourth digit refer to specific DRGs groups called the Case-Based Group (CBG), and the final digit refers to the patients’ severity level (I = mild, II = moderate, III = Severe) (Fig. 2).

The accuracy of the coding is crucial, especially when the hospital is using the Casemix System as their provider payment tools. However, the coding process is an error-prone, and any error may lead to far-reaching consequences. Coding error is a condition in which the codes assigned by in-house medical coders differs from those assigned by the independent reviewer during the coding audit [10]. A wrong code will relate to an incorrect assignment of the DRGs code, and it may have an adverse impact on hospital income in countries that used Casemix System as their provider payment tools [11]. Thus, the aim of this study is to analyse coding error during the implementation of the Malaysia Diagnostic Related Group (MY-DRG^®^) Casemix System in a teaching hospital in Malaysia and its impact on potential hospital revenue.

## Methods

### Study design and population

This study was focused on the impact of the coding errors on potential hospital revenue. A cross sectional descriptive study was conducted in one of Malaysia’s teaching hospital from January to December of 2013. A total of four hundred and sixty four (464) of patient medical records from 4 disciplines; Medical, Surgical, Obstetric & Gynecology (O&G) and Paediatric were sampled from the year 2013. These selected records were stratified according to the four disciplines and were randomly selected according to the sample requirement. Selected records were ensured to be coded beforehand by the clinical coder based in the clinical coding department in this teaching hospital.

### Audit process

For the clinical coding audit purposes, an independent senior coder (not employed by the hospital) was appointed to review and re-code the diagnosis and procedure code of the selected records. The independent senior coder reviewed the entire case notes and all aspects of the clinical coding were reviewed and evaluated. The independent senior coder also reviewed and evaluated the completeness of the documentation for each selected records. This independent senior coder has more than 30 years of experience in coding and has undergone coding courses and training at the international level. During the early implementation and establishment of the coding unit in this hospital, this independent senior coder was appointed as the coding trainer to train the in-house clinical coders. This independent senior coder is also actively involved in training coders from public and private hospital in Malaysia.

After the re-coding process was completed, the new codes assigned by the independent senior coder and the old codes were compared. If they differed, then the new codes assigned by the independent senior coder were considered as the accurate codes. The new codes assigned by the independent senior coder were then reviewed and verified by the Casemix Experts. If the experts approved the code, it was then used, and the discharges were re-grouped again using the MY-DRG^®^ grouper to identify the new MY-DRG^®^ code. Lastly, this new MY-DRG^®^ code was compared with the original MY-DRG^®^ code to identify if there were any changes in the assignment of the hospital tariff. These Casemix Experts are the researchers who were involved in the implementation of Casemix system in Malaysia and they are also the developers of MY-DRG^®^ grouper employed by this hospital.

### Statistical analyses

A descriptive analysis was used to measure the percentage of coding error occurred during the study. The calculation of the coding error percentage was conducted by using the total number of cases with errors as the numerator and the total number of reviewed cases as the denominator.

In the bivariate analysis, a Chi-square test was employed to identify the association between coding error and the type of discipline and patient severity level. Cohen’s Kappa test was used to evaluate the level of agreement in the assignment of every coding item before and after the re-coding process; it included primary diagnosis code, secondary diagnosis code, primary procedure code and secondary diagnosis code. The Cohen’s Kappa test was also used to identify the level of agreement in the assignment of the MY-DRG^®^ code before and after the re-coding process. Kappa values were interpreted as follows;

i. 0.01 to 0.20 = poor agreement
ii. 0.21 to 0.40 = fair agreement
iii. 0.41 to 0.60 = moderate agreement
iv. 0.61 to 0.80 = substantial agreement
v. 0.81 to 1.00 = perfect agreement

A multiple logistic regression was employed to predict the factors that influenced the coding error. This test was applied by using the type of case (with coding errors or without coding errors) as the dependent variable and type of disciplines, completeness of admission forms, completeness of discharge summaries, type of severity level, and coder demographic as the independent variable.

The assignment of hospital tariff for the selected cases was performed according to the hospital tariff per MY-DRG^®^ code as calculated by the Casemix Expert based in the Casemix Centre of this hospital. A descriptive analysis was conducted to measure the total potential revenue, mean revenue, minimum revenue and maximum revenue before and after the re-coding process. The statistical significance of the differences in the total potential of revenue before and after the re-coding process was measured using a paired sample t-test in which the test was conducted by comparing the original potential revenue against new potential revenue.

The association between the coding error and the hospital’s potential revenue was determined using the multiple logistic regressions. The type of coding error (errors in the primary diagnosis code, secondary diagnosis code, primary procedure code, and the secondary procedure code), type of discipline, type of severity, completeness of the discharge summary, completeness of the admission form and demographic of the coders were analysed to determine the effects of these variable on the accuracy of the assignment of hospital tariff.

All the data analyses were done using IBM SPSS version 20.0 and a *p*-value of *p* <  0.05 was considered as statistically significant.

## Results

### Coding error rates

The findings from the re-coding process by the independent senior coder revealed that a total of 415 (89.4%) of the selected patient medical record contained at least one difference in their diagnosis or procedure code. Table 1 illustrates the overview of the distributions in coding error cases that occurred in this study.

**Table 1:** Coding error rate by discipline and severity level

Item reviewed	Total case reviewed	Error case (%)	Chi square	*p* value
Type of Discipline			11.515	0.009
Medical	116	107 (92.2)		
Surgical	116	96 (82.8)		
O&G	116	110 (94.8)		
Peadiatric	116	102 (87.9)		
Type of Severity Level			17.658	< 0.001
I	297	259 (87.2)		
II	124	115 (92.7)		
III	43	41 (95.3)		

From Table 1 it is aparent that the coding error cases were exceptionally high in the O&G discipline covering 110 (94.8%) of the selected cases. The second-highest number of coding error cases were found in the medical discipline, with a total of 107 (92.2%) error cases. The paediatric discipline had the third-highest number of coding errors case whereby the total number of error cases was 102 (87.9%) cases. The surgical discipline reported fewer coding error cases with the number of error cases was 96 (82.8%) cases, respectively (Table 1). The difference between the percentage of coding erros by the type of discipline was statistically significant with chi square value of *X*^2^ (3) = 11.518, *p* = 0.009.

This study also revealed that the coding error cases were more common among severity level III cases than the lower severity cases. The total number of coding error cases for severity level III was 41 (95.3%) cases (Table 1). The difference of the coding error rate between severity levels was proven to be statistically significant at *X*^*2*^ (2) = 17.658, *p* <  0.001.

### Coding errors by coding items

After the re-coding process by the independent senior coder, coding error cases were commonly found during the assignment of the secondary diagnosis code covering 377 (81.3%) of the cases. The level of agreement between the independent senior coder and the original coder during the assignment of the secondary diagnosis code was poor, with a kappa value of 0.108. Findings from the re-coding process indicated that the primary diagnosis code contained fewer coding error cases in which the total number of coding error cases reached 231 (49.8%) cases. The level of agreement between the independent senior coder and the original coder during the assignment of the primary diagnosis code was moderate with a kappa value of 0.495.

Coding error involving primary diagnosis codes were the highest among the medical discipline, with a total number of coding error cases of 65 (56.0%) (k = 0.441). By contrast, coding errors involving a primary diagnosis code were the lowest within the surgical discipline, in which the coding error cases reached only 47 (40.5%) cases (k = 0.581).

During the assignment of secondary diagnosis codes, the O&G discipline showed the highest percentage of coding error cases with a total error cases, with a total of 108 (95.1%) cases (k = 0.038). Contrary, the paediatric discipline showed the lowest percentage of coding error cases in the assignment of secondary diagnosis code with a total of 84 (72.4%) cases (k = 0.261).

The top discipline containing coding error cases during the assignment of primary procedure codes was the medical discipline for a total of 66 (56.9%) coding error cases (k = 0.010). On the other hand, the coding error cases among primary procedure codes were the lowest within the O&G discipline with a total of 51 (44.0%) cases (k = 0.023).

During the assignment of secondary procedure code, the O&G discipline showed a higher percentage of coding error cases. After the re-coding process by the independent senior coder, the number of coding error cases among O&G cases was 98 (84.5%) cases (k = 0.052). Among these four disciplines, the paediatric discipline showed the lowest percentage of coding error cases with a total number of 29 (52.6%) cases (k = 0.202). Table 2 illustrates an overview of coding error cases per coding item.

**Table 2:** Coding error rate by coding items

Coding items	Total case reviewed	Total error cases (%)	Kappa value	*p* value
Primary diagnosis code				
All Cases	464	231 (49.8)	0.495	< 0.001
Medical	116	65 (56.0)	0.441	< 0.001
Surgical	116	47 (40.5)	0.581	< 0.001
O&G	116	61 (52.6)	0.444	< 0.001
Paediatric	116	58 (50.0)	0.460	< 0.001
Secondary diagnosis code				
All Cases	464	377 (81.3)	0.108	< 0.001
Medical	116	98 (84.5)	0.180	< 0.001
Surgical	116	87 (75.0)	0.171	< 0.001
O&G	116	108 (93.1)	0.038	< 0.001
Paediatric	116	84 (72.4)	0.261	< 0.001
Primary procedure code				
All Cases	464	236 (50.9)	0.159	< 0.001
Medical	116	66 (56.9)	0.010	< 0.001
Surgical	116	58 (50.0)	0.022	< 0.001
O&G	116	51 (44.0)	0.023	< 0.001
Peadiatric	116	61 (52.6)	0.013	< 0.001
Secondary procedure code				
All Cases	464	270 (58.2)	0.210	< 0.001
Medical	116	67 (57.8)	0.176	< 0.001
Surgical	116	76 (65.5)	0.145	< 0.001
O&G	116	98 (84.5)	0.052	< 0.001
Peadiatric	116	29 (25.0)	0.202	< 0.001

### Coding error cases with changes in the assignment of MY-DRG^®^ code

After the re-coding process, a total of 307 (74.0%) error cases showed changes in the assignment of MY-DRG^®^ codes. During the re-assignment of MY-DRG^®^ codes, the largest changes were involving the third digit level of the MY-DRG^®^ code nameley the DRGs group in which 131 (42.7%) of the cases were affected (k = 0.284). Findings from the re-coding process revealed that the assignment of the second digit level of MY-DRG^®^ code nameley the assignment of the discipline was less affected by the coding errors. Out of 307 cases with changes in the MY-DRG^®^ code, there were only 12 (3.9%) cases that were reported to have changes in the discipline assignment (k = 0.826). Table 3 summarises the changes in the assignment of MY-DRG^®^ codes due to the coding errors.

**Table 3:** Changes in the assignment of MY-DRG^®^ codes

Item	Nos cases (%)*	Kappa value	*P* value
Changes in CMG	69 (22.5)	0.823	< 0.001
Changes in Discipline	12 (3.9)	0.826	< 0.001
Changes in DRG Group	131 (42.7)	0.284	< 0.001
Changes in Severity Level	87 (28.3)	0.489	< 0.001
Ungroupable	8 (2.6)	N/A	N/A

*n = 307

Apparently from Table 3, after the re-coding process, there were 8 (2.6%) ungroupable cases recorded in this study. These ungroupable cases were originally assigned to paediatric discipline and interestingly the birth weight could for all these 8 cases were missing from the patient’s case notes as well as in the discharge summary.

### Factors influencing the coding errors

The findings from the multivariate analysis indicate that the type of discipline, the completeness of the discharge summary and the completeness of the admission form could influence the coding errors.

As shown in Table 4, the completeness of the discharge summary and the completeness of the admission form was the most important factor that influenced the coding errors. A case with an incomplete admission form was 215 times higher (OR = 215.04, 95% Confidence limits = 48.096 – 962.022) to be coded incorrectly than cases with complete admission form. On the other hand, a case with an incomplete discharge summary was 143 times more likely (OR = 143.056, 95% Confidence limits = 40.323 – 507.521) to be coded incorrectly than cases with complete discharge summary.

**Table 4:** Factors influencing coding errors

Variables	B	S.E	*p* value	Wald	OR	95% Confidence limits
Disciplines						
Medical	1.043	0.441	0.018	5.594	2.839	1.196 - 6.739
O&G	1.340	0.486	0.006	7.604	3.819	1.473 - 9.901
Paediatric	0.329	0.370	0.375	0.787	1.389	0.672 - 2.870
Incomplete Admission Form	5.371	0.764	< 0.001	49.391	215.014	48.096 - 962.022
Incomplete Discharge Summary	5.369	0.764	< 0.001	49.346	143.056	40.323 - 507.521
Severity Levels						
II	0.628	0.387	0.105	2.663	1.875	0.878 - 4.005
III	1.101	0.745	0.139	2.187	3.008	0.699 - 12.946
Coders’more and equals to 10 years Lengths of Service	0.322	0.405	0.427	0.631	1.379	0.624–3.049
Coders’ with less than 5 Coding Trainings Session	0.490	0.313	0.117	2.453	1.632	0.884 - 3.015
Non-Degree Coders	−0.322	0.405	0.427	0.631	0.725	0.328–1.603

 During the bivariate analysis, it was revealed that the lowest coding errors rate was among the surgical cases. Subsequently, in the multivariate analysis using the multiple logistic regression, it was discovered that an O&G case is about four times more likely (OR = 3.819, 95% Confidence limits = 1.473 - 9.901) to be coded wrongly than a surgical case. On the other hand, a medical case is about 3 times more likely (OR = 2.838, 95% Confidence limits = 1.196 - 6.739) to be coded wrongly than a surgical case. Interestingly as notable in Table 4, the possibilities of t a paediatric case to be coded wrongly than surgical case could not be determined statistically (*p*>0.005). This is due to the insignificant difference of coding errors rate among surgical and paediatric disciplines.

This study also evaluated the type of severity levels and coders’ demographic to determine their association with coding errors. However, the output from the statistical analysis showed that in this study, the coding errors are unassociated with these factors.

### Impact of coding error on potential hospital revenue

This finding indicates that the coding errors that occurred in the hospitals were more likely related to profit loss than profit gain. Among the 307 coding error cases with changes in the MY-DRGs code assignments, 52.1% (160/307) of the cases were assigned a lower hospital tariff. Table 5 shows the overview of the potential revenue before and after the re-coding process.

**Table 5:** Overview of hospital potential revenue before and after the re-coding process

		Total revenue**	Mean**	Minimum**	Maximum**	t value	*p* value
All Cases	Before	1,672,922.13	3508.45	1020.24	39,993.00	−2.648	0.008
	After	2,327,226.07	5015.57	0.00	233,318.94		
Medical	Before	351,450.99	3003.85	1020.24	15,836.25	−3.044	0.003
	After	437,255.91	3363.51	1160.19	24,547.17		
Surgical	Before	619,472.01	5340.27	1530.00	39,993.00	0.753	0.453
	After	625,701.30	6257.01	1524.42	30,273.31		
O&G	Before	395,988.45	3413.69	2614.74	14,767.00	−0.768	0.444
	After	414,226.06	3480.89	2614.74	14,767.00		
Paediatric	Before	261,010.68	2269.66	1412.00	6098.51	−2.398	0.018
	After	850,042.00	7798.56	1343.00	233,318.97		
Ungroupable Cases^*^	Before	20,773.00	3462.17	1412.00	5518.00	5.945	0.002
	After	0.00	0.00	0.00	0.00		

*All ungroupable cases were originally grouped under Paediatric Discipline

**All amount are shown in Malaysian Ringgit (MYR)

As apparent in Table 5, the potential hospital revenue that this hospital could gain in the coding error cases was RM1,672,922.13 with a mean revenue of RM3508.45. However, without the coding error cases, this hospital could potentially gain RM2,327,2267.07 with a mean revenue of RM 5015.57. Before the re-coding process, the minimum potential revenue for this hospital was RM1020.24. However, due to the ungroupable cases, these cases could not be assigned to any hospital tariff, and they caused the minimum potential revenue to decrease sharply to RM0.00. Even though the minimum potential income dropped sharply after the re-coding process, the maximum potential income after the re-coding process increased to 82.8% higher than it was before the re-coding process. The maximum potential income before the re-coding process was RM39,993.00, and it rapidly increased to RM233,318.94 after the re-coding process. The difference in the total potential revenue was shown to be statistically significant (t (463) = − 2.648, *p* = 0.008).

After the re-coding process, the paediatric discipline showed the biggest variation in potential revenue. Before the re-coding process, the total potential revenue under this discipline was RM261,010.68, and it greatly increased to RM850,042.04 after the re-coding process by the independent coder. One plausible explanation for this large variation was due to the changes in the hospital tariff for one episode of care from RM 3292.00 before the re-coding process to RM233,319.00 after the re-coding process. Another striking finding in the assignment of hospital tariffs within the pediatric discipline was that the minimum potential revenue decreased from RM1412.00 before the re-coding process to RM1343.00 after the re-coding process by the independent coder. The difference in total potential revenue before and after the re-coding process within the paediatric discipline was statistically significant (t (115) = − 2.398, *p* = 0.018).

During the calculation of the hospital’s potential revenue, the lowest profit gain was among the O&G discipline. A total of RM395,988.45 in potential income was recorded before the re-coding process, and this number slighly increased to RM 414,226.00 after the re-coding process. The minimum potential income and maximum potential income within the O&G discipline showed a same amount before and after the re-coding process. Subsequently, even though there was a difference in the total potential revenue within the O&G discipline, the difference was identified to be statistically insignificant (t (115) = − 0.768, *p* = 0.444).

Another important finding in this study involved the ungroupable cases. After the re-coding process, 8 of the ungroupable cases were assigned to a hospital tariff amounting to RM0.00. However, these cases were initially grouped into the paediatric discipline, and the minimum hospital tariff that the hospital could assign among these 8 cases was RM1412.00. The documentation issue that relates to the coding errors caused this hospital to face profit losses from these ungroupable cases. The changes in the assignment of hospital tariffs within these ungroupable cases were shown to be statistically significant (t (7) = 5.945, *p* = 0.002).

In using Casemix system as the provider payment tools, the loss of income is associated with the wrong assignment of hospital tariff per patient. Table 6 shows the factors that could potentially cause wrong assignment of hospital tariff. As visible in Table 6, this study indicates that all factors listed in Table 6 excluding error cases of primary procedure code could contribute to changes in the assignment of hospital tariffs. The most influential factor was a case with an error in the assignment of its severity level. The output from the multiple logistic regression indicates that a case with errors in the assignment of its severity level is 65 times more likely (OR = 65.416, 95% Confidence limits = 23.601 - 181.314) to be assigned to an inaccurate hospital tariff compared to a case without error in the assignment its severity level.

**Table 6:** Factors influencing accuracy of the assignment of hospital tariff

Variables	B	S.E	*p* value	Wald	OR	95% Confidence limits
Coding Error of Primary Diagnosis Code	0.798	0.201	< 0.001	15.804	2.221	1.499 - 3.292
Coding Error of Secondary Diagnosis Code	1.698	0.255	< 0.001	44.210	5.465	3.313–9.017
Coding Error of Primary Procedure Code	0.156	0.274	0.570	0.322	1.168	0.683 - 1.999
Coding Error of Secondary Procedure Code	0.568	0.198	0.004	8.221	1.764	0.197 - 2.601
Coding Error with the Error in the Assignment of Severity Level	4.181	0.520	<0.001	64.604	65.416	23.601 - 181.314
Coding Error with Incomplete Admission Form	2.026	0.474	< 0.001	18.252	7.583	2.994 - 19.209
Coding Error with Incomplete Discharge Summary	2.023	0.448	< 0.001	17.362	6.478	2.690 - 15.601

The second most influential factor contributing to error in the assignment of hospital tariff was coding error case of secondary diagnosis code. From Table 6, it is notable that a case with errors of secondary diagnosis code is 5 times more likely (OR = 5.465, 95% Confidence limits = 3.313 - 9.017) to be assigned to an inaccurate hospital tariff compared to a case without error of secondary diagnosis code.

## Discussion

This study showed that the coding error rate in this hospital is very high, at 89.4% (*n* = 415/464) of the selected cases. In comparing it with other studies using the same methodology, the percentage of coding error is very high. For example, in a study conducted in Saudi Arabia, only 30.0% of the coding error cases were reported after the coding audit was complete [12]. However, this previous study employed a physician to conduct the coding audit. In contrast, in this study, the audit was performed by an independent senior coder. Physicians and senior coders may have different views about the assignment of diagnosis and procedure codes. Even though the physician is an expert in determining the patient’s diagnostic and procedural phases, their knowledge and experience in the assignment of diagnosis and procedure codes could be limited. By contrast, an expert senior coders will have broader experience and knowledge in the coding field, which will lead them to a more detailed review during the audit; this experience explained the higher coding error rate in this study. In a previous study conducted in the United Kingdom, the overall coding error rate recorded for the study was 51.0% [13]. The overall percentage of the coding error is lower than it was in this hospital, but this study reported the coding error rate for the surgical department only.

Findings from this study also highlighted that most of the errors were primarily found in the secondary diagnosis, at 81.3% (*n* = 377/464). This finding is consistent with past study findings from Saudi Arabia and Thailand, which reported a higher coding error rate in secondary diagnosis (35.6% and 28.0%) [12, 14]. In the coding rules, a physician could assign up to 29 secondary diagnostic phrases for each patient. The higher the number of secondary diagnosis phrases there are, the higher the chances the case will be coded incorrectly by the coder. Without proper training and knowledge, a higher number of secondary diagnosis phrases will cause confusion and difficulties for coders in assigning the appropriate codes for each diagnosis. However, this result is contrary to that of a study conducted within an Otolaryngology department in the United Kingdom. That study reports a coding error that is higher in the procedure code (14.9%) [15]. A plausible explanation for this contradiction is that the study was conducted in a department where the type of procedures assigned to the selected cases may be more complicated than those of our selected patient medical records.

Coding errors will lead to far-reaching consequences in hospital incomes, especially when the hospital uses a casemix system as their provider payment tool [16, 17]. Consistent with findings by Curtis et al. and Nouraei et al., this study showed that coding error will lead to more loss rather than profit gain [18, 19] . This study recorded a total potential loss of RM654,303.91, which is equivalent to 39.1% of the potential hospital income. Curtis et al. also reported the same scenario in their study in which the coding error in a trauma patient’s clinic caused a total loss of RM117,464.00 (AUD 39960). Nouraei et al. also reported that coding errors (184 cases) led to lower hospital tariffs before the clinical audit was conducted.

Despite prior studies reporting that the coding error was higher in the surgical discipline [12, 20], the findings from this study revealed that the coding error was deemed higher among O&G cases (84.5%). This is due to the high percentage of error in the secondary diagnosis code and secondary procedure code within the O&G discipline. However, even though the coding error rate in the O&G discipline was extensive, the potential profit loss in total hospital income (RM14,825.39) was statistically insignificant (*p* <  0.444). The minimum hospital tariff that could possibly be assigned to a patient after the re-coding process was in accordance with the minimum hospital tariff before the re-coding process (RM 2614.00). The maximum hospital tariff among O&G cases was also unaffected due to the coding errors in which the maximum hospital tariff was RM 14,767.00 before and after the re-coding process. The most common changes detected during the assignment of the MY-DRG^®^ code involved the changes in the MY-DRG^®^ code from O-6-12-I (Vaginal Delivery With Other Procedure Excluding Sterilization &/Or Dilation & Curettage) to O-6-13-I (Vaginal Delivery, Mild) to the increase in the tariff from RM 2614.74 to RM 2820.56. There were a total of 9 cases with this type of change after the re-coding process by the independent coder.

This study indicated that the potential profit loss from the medical discipline was RM85,804.92. Before the re-coding process by the independent senior coder, the minimum hospital tariff assigned to patients under this discipline was RM1020.24 and it increased to RM1160.19 after the re-coding process. The maximum potential hospital tariff under this discipline also has also a significant improvement from RM15,836.00 to RM24,547,17. From the data analysis, it was found that the highest potential profit loss from the medical discipline was RM24,250.00. This finding is due to the change in the patient MY-DRG assignment from B-4-14-II (Other Biliary Tract Diseases - Moderate) to B-1-10-III (Pancreas & Liver Operations) when the former tariff was RM2,299.00 and the updated one was RM26,549.00. The coding errors detected within this case contained major errors as related to changes in the discipline assignment of the case.

From this study’s findings, the potential hospital revenue under the paediatric discipline has increased rapidly from RM261,010.68 to RM850,042.00. This findings shows a greater than 200% increase from before the re-coding process. Among the plausible explanations for this finding is that under this discipline, there were eight error cases, relating to potential profit losses of more than RM20,000.00. The most noticeable loss was the case with a change from MY-DRG code P-8-17-III (Neonate, Birth-weight > 2499 g Without Complex Operation-Mild) to P-8-02-III (Neonate, Birth-weight < 1000 g With Complex Operation – Major). The original hospital tariff for this case was only RM3292.00. However, after the re-coding process by the senior coder, the new hospital tariff grew to RM233,319.00. This huge variance in the hospital tariff was due to the coding errors in the assignment of the primary and secondary diagnosis codes as well as the disparity of birth-weight information between the patient’s case notes and the discharge summary. To eliminate coding errors cases involving the poor quality of discharge summary, the coding process conducted in this hospital should be performed according to the hospital’s coding guidelines (coding based on the patients’ case notes).

The data analysis from this study shows that the completeness of the admission form (*p* <  0.001) and the completeness of the discharge summary (*p* <  0.001) will have an impact on coding errors as the coding process conducted by the original coder in this hospital was performed according to the admission form and discharge summary. The more incomplete or inaccurate is the documentation, the more likely the case will have error in the diagnosis or procedure codes. Other study have also reported that the accuracy and completeness of the documentation is highly important to avoid any errors during the coding process [21, 22]. O’Malley also stressed in his study that the quality of information during admission is crucial in preventing the hospital from facing any losses due to the coding errors [23].

This coding error incidence will not only affect the hospital but will also have substantial consequences for the patient. Jesilow reported in his study that the coding error has caused patient to pay twice from the actual treatment cost [24]. For example, this study indicates coding errors cases within the surgical discipline in relation to profit gains. According to the correct codes assigned by the independent coder, the maximum hospital tariff that could be charged to the patient within this discipline was RM30,273.31. However, with the current coding quality, the maximum hospital tariff that this discipline could charge the patient was RM 39,993.00. Even though the current coding quality within surgical discipline contributes to profit gains for the hospital, the cost incurred by the patient was not the actual cost of the treatment and caused the patient to pay higher than the actual cost of the treatment.

This study only undertook codes from medical records in 2013, and only in-patient cases were selected for the re-coding process. The coding error rate may decrease from year to year as the coders in this hospital are increasingly exposed to the coding guideline and process.

## Conclusions

This study revealed that the coding error rate in this hospital is very high, especially for the assignment of secondary diagnosis codes. The O&G discipline has shown the highest coding error rates compared to other disciplines. Thus, it is recommended that the coders pay more attention during the assignment of O&G cases, especially during the assignment of secondary diagnosis codes. A continuous coding training should be undertaken to ensure that the coders’ skills are aligned with the current coding rules and guidelines.

This study also indicates the importance of following the coding guidelines during the coding process. The coding error rate detected in this study could be reduced if the coders referred to the entire case note for the episode of care during the coding process. Conducting a coding process by referring to the admission form and discharge summary alone is insufficient for this hospital because the quality of these documents is very poor. An improvement in the documentation is highly necessary if the coders would like to conduct the coding process without analyzing the entire case notes.

Certainly, good coding skill and knowledge among coders are crucial in the effort to decrease the coding error rate. However, physician involvement in ensuring better documentation in the admission form and discharge summary is also needed to reduce the coding error rate in this hospital. Accurate documentation in terms of the admission form and discharge summary plays a significant role in helping the coders to code all the cases accurately. This study suggests that highly skilled physicians and coders are the key to ensuring high-quality coded data. Good cooperation and communication between coders and physicians must occur to provide a lower coding error rate in the hospital.

Further research involving different institutes is needed, not only to support the findings of this study but also to help improve Malaysia’s healthcare regarding their distribution of financial resources by using high-quality coded data. In addition, future research about physician influence on the accuracy of the coding could also be conducted because this study only focuses on the influence of the coders on the coding accuracy.

## Abbreviations

MY-DRG^®^: Malaysia Diagnostic Related Group
O&G: Obstetric & Gynecology
